# Global gene expression profile of cerebral ischemia-reperfusion injury in rat MCAO model

**DOI:** 10.18632/oncotarget.20253

**Published:** 2017-08-14

**Authors:** Chunmei Wang, Minghui Liu, Yanyou Pan, Bo Bai, Jing Chen

**Affiliations:** ^1^ Neurobiology Institute, Jining Medical University, Jining 272067, P.R. China; ^2^ Division of Biomedical Sciences, Warwick Medical School, University of Warwick, Coventry CV4 7AL, U.K

**Keywords:** gene expression profile, ischemia-reperfusion injury, MCAO model, RNA sequencing

## Abstract

It is well-established that reperfusion following cerebral ischemic injury gives rise to secondary injury accompanied by structural and functional damage. However, it remains unclear how global genes changes in cerebral ischemia-reperfusion injury (IRI). This study investigated global gene expression in the hippocampi of Wistar rats following transient cerebral IRI using an RNA-sequencing strategy. The results revealed ≥2-fold up-regulation of 156 genes and ≥2-fold down-regulation of 26 genes at 24 h post-reperfusion. Fifteen differentially expressed genes were selected to confirm the RNA-sequencing results. Gene expression levels were dynamic, with the peak expression level of each gene occurring at different time points post-reperfusion. Gene Ontology (GO) analysis classified the differentially expressed genes as mainly involved in inflammation, stress and immune response, glucose metabolism, proapoptosis, antiapoptosis, and biological processes. KEGG pathway analysis suggested that IRI activated different signaling pathways, including focal adhesion, regulation of actin cytoskeleton, cytokine-cytokine receptor interaction, MAPK signaling, and Jak-STAT signaling. This study describes global gene expression profiles in the hippocampi of Wistar rats using the middle cerebral artery occlusion (MCAO) model. These findings provide new insights into the molecular pathogenesis of IRI and potential drug targets for the prevention and treatment of IRI in the future.

## INTRODUCTION

Ischemic stroke is one of the leading causes of death and disability worldwide, making its prevention and treatment urgent. Evidence from clinical trials suggests that the most effective treatment currently available is thrombolysis therapy [1, 2]. However, reperfusion after thrombolysis may further exacerbate cerebral ischemic injury by disrupting cerebral microvascular endothelial cells, a phenomenon referred to as cerebral ischemia-reperfusion injury (IRI) [3, 4]. Currently, there is no approved therapy for the treatment of IRI.

The mechanisms of cerebral IRI are complex. During reperfusion, blood flow is reintroduced to the tissue, triggering a series of events including oxidative stress, energy failure, inflammation, excitatory amino acids toxicity, the activation of various cell-signaling pathways, apoptosis, and neuronal death [5, 6]. Meanwhile, the ischemia-reperfusion process usually lead to increase or decrease of a number of genes, such as insulin-like growth factor-1 (IGF-1)[7], heat shock protein (HSP27) [8], protein tyrosine kinase type 2 beta (Ptk2b) [9], high mobility group box 1 (HMGB1) [10], and pyruvate dehydrogenase kinase 4 (PDK4) [11]. We hypothesize that the process of reperfusion induces the abnormal expression of a large number of genes. This altered gene expression might activate or suppress corresponding signaling pathways, resulting in cellular necrosis or apoptosis.

In the present study, we describe changes in global gene expression in rat hippocampi after cerebral IRI. These results provide new insights into understanding the mechanisms of cerebral IRI. Furthermore, the identified genes may be considered potential drug targets for the prevention and treatment of cerebral IRI.

## RESULTS

### IRI induces cerebral infarct

**Figure 1:**
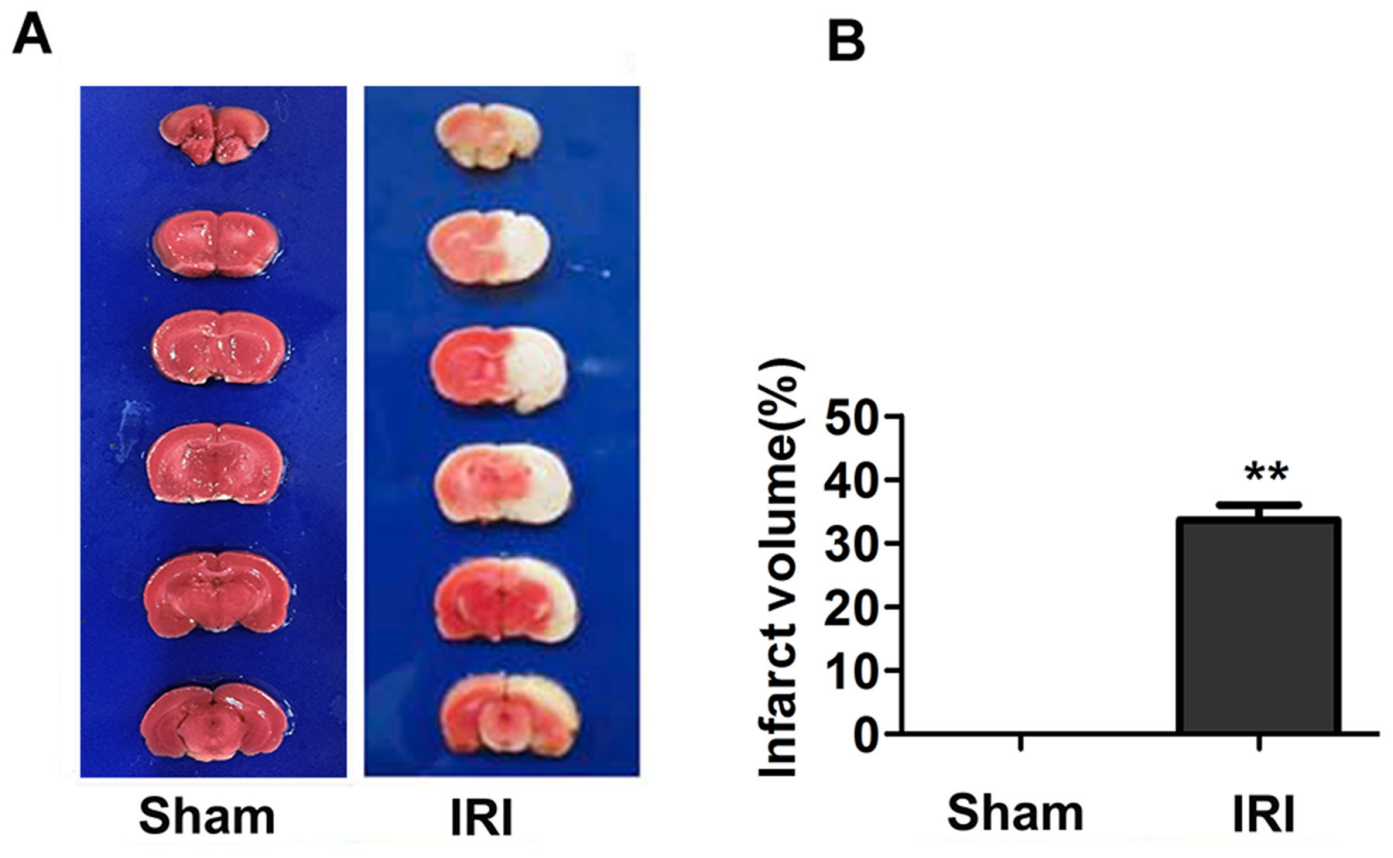
Infarct volume in sham and IRI/24 h groups determined by TTC staining (Panel **A**) Representative coronal brain sections of the sham and IRI/24 h groups. Brain sections in the sham group showed no infarction. The infarct volumes in the IRI/24 h group were significantly increased compared with those in the sham group. (Panel **B**) Percentage of cerebral infarct volume to total brain volume. The percentage of infarct volume was approximately 33.6% at 24 h in the IRI model. **P < 0.01.

The infarct areas appeared white when stained with TTC. As shown in Figure 1, brain tissue in the sham group appeared uniform red in color, indicating no infarction. After the right cerebral hemisphere was subjected to ischemia and reperfusion, the infarct volume was increased 33.64% ± 2.06% compared with the sham group. This result demonstrated that IRI induced cerebral infarct, indicating that the model was successful.

### Global gene expression profile of IRI

**Figure 2:**
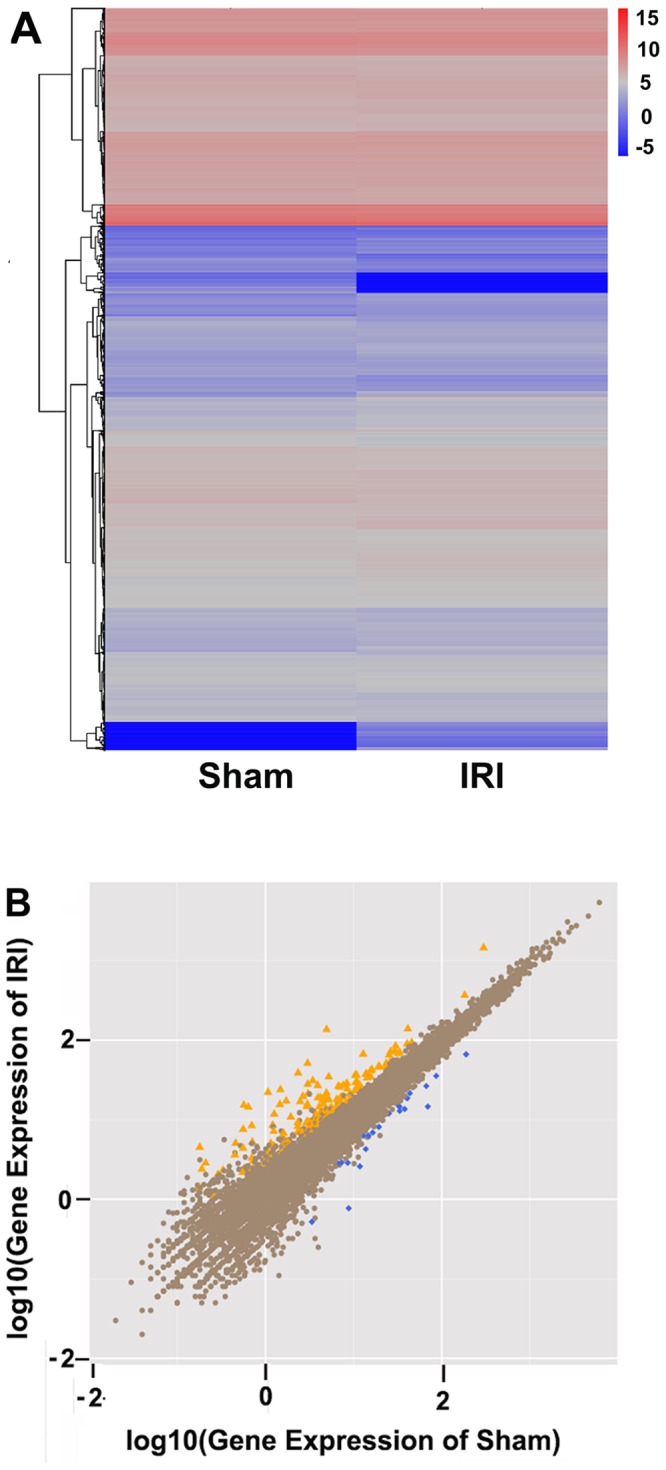
Comparison between sham and IRI/24 h groups by RNA sequencing (Panel **A**) The differentially expressed genes between the sham group (n = 6) and the IRI/24 h group (n = 6) were analyzed using SAM software and visualized with Treeview tools upon hierarchical clustering. A colored tag represents gene expression levels. Red spectrum denotes up-regulated genes, and blue denotes down-regulated genes. (Panel **B**) Scatter plot display of the differentially expressed genes between sham and IRI groups. The X and Y axes represent the log2 fold change in gene expression. Yellow points represent the up-regulated genes with fold change less than 1 and *P* < 0.05. Blue points denote the down-regulated genes with fold change greater than 1 and *P* < 0.05. Brown points indicate genes in which there were no significant differences in expression.

A global gene expression profile provides comprehensive insights into which hippocampal genes participate in the pathology of cerebral IRI. Genes with similar expression patterns often have a functional correlation. We carried out cluster analysis of gene expression patterns using cluster and java Treeview software. There were significant differences in cluster analysis between the sham group and the IRI/24 h group (Figure 2A). The genes with increased expression were divided into four groups. Genes with decreased expression were divided into six groups.

182 genes demonstrated differential expression in the IRI/24 h group relative to the sham group. As shown in Figure 2B, 156 genes were significantly up-regulated in the IRI/24 h group when compared with the sham group, and 26 genes were significantly down-regulated. There was a more than 2-fold change in expression levels of the indicated genes in the IRI/24 h group relative to the sham group. It is likely that these 182 genes involve in the pathology of IRI, and their annotation will help to further elaborate the molecular mechanism of IRI. A portion of the differentially expressed genes are listed in Table 1.

**Table 1:** Genes differentially expressed between sham and IRI/24 h groups

Gene ID	Description	Symbol	Fold change
24471	heat shock protein B1	Hspb1	10.84648
24498	interleukin 6	IL-6	10.40939
25544	selectin E	Sele	7.965784
89829	suppressor of cytokine signaling 3	Socs3	7.267831
307351	tubulin, beta 6 class V	Tubb6	6.173174
24472	heat shock 70 kD protein 1A	Hspa1a	4.493855
24577	myelocytomatosis oncogene	Myc	3.284498
680611	B-cell CLL/lymphoma 3	Bcl3	2.885765
24887	Bcl2-associated X protein	Bax	2.819229
314322	FBJ osteosarcoma oncogene	Fos	2.981011
304962	activating transcription factor 6	Atf6	2.781895
24387	glial fibrillary acidic protein	Gfap	2.479572
24451	heme oxygenase (decycling) 1	Hmox1	2.285812
308995	integrin, alpha L	Itgal	2.165753
361321	autophagy related 12	Atg12	3.980192
84398	CD93 molecule	Cd93	3.581731
60350	CD14 molecule	CD14	2.475128
83785	vascular endothelial growth factor A	Vegfa	2.773833
497942	chemokine ligand 16	Cxcl16	2.54374
315609	CD3 molecule, epsilon	Cd3e	-6.21785
292843	sialic acid-binding Ig-like lectin 5	Siglec5	-5.69617
299757	neurotensin	Nts	-4.54653
83725	Wolfram syndrome 1 (wolframin)	Wfs1	-2.85056
24224	B-cell CLL/lymphoma 2	Bcl-2	-1.68324

### Confirmation of differentially expressed genes by RT-PCR

**Figure 3:**
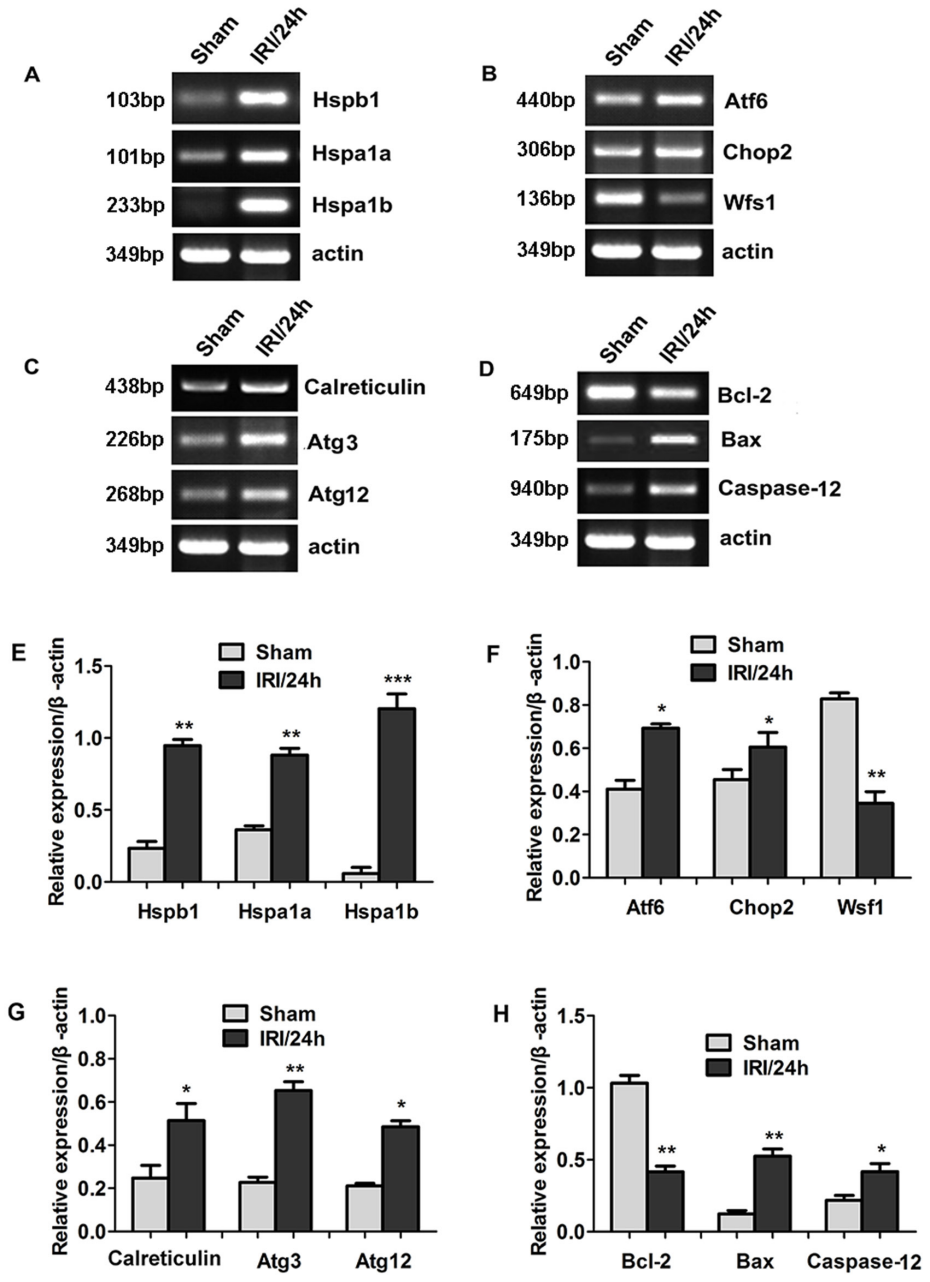
Differential gene expression between sham and IRI/24 h groups was detected by RNA sequencing (Panel **A-D**) Representative images of RT-PCR confirming differential gene expression. (Panel **E-H**) Quantitative analysis of gene expression. *P < 0.05, **P < 0.01, ***P < 0.001 *vs* sham.

To confirm the results obtained from RNA sequencing, we examined the expression of representative genes at the mRNA level using RT-PCR (Figure 3). The sequences of all primers used are listed in Tables 2 and 3. The genes belonging to the heat shock protein (Hsp) family were selected for amplification because their expression showed the most significant up-regulation in the RNA-sequencing studies. As shown in Figure 3A, expression of Hspb1, Hspa1a, and Hspa1b was significantly increased in the IRI/24 h group compared with the sham group with a fold change of 4.06, 2.43, and 20.88, respectively (Figure 3E, **P < 0.01, ***P < 0.001). Two genes, Atf6 and Chop2, related to endoplasmic reticulum (ER) stress, were also significantly up-regulated in IRI/24 h (Figure 3B). The fold change in the IRI/24 h group relative to the sham group of Atf6 and Chop2 was 1.69 and 1.33, respectively (Figure 3F, *P < 0.05). The expression of Wfs1, another gene related to ER stress, was down-regulated 2.4-fold (Figure 3F, **P < 0.01) in the IRI/24 h group. Three genes, calreticulin, Atg3, and Atg12, were related to autophagy. Their relative mRNA levels were significantly higher with fold changes of 2.13, 3.25, and 2.36, respectively. (Figure 3C and 3G, *P < 0.05, **P < 0.01). The expression of Bcl-2 was lower, while the mRNA levels of Bax and Caspase-12 were higher, in the IRI/24 h group than in the sham group (Figure 3D and 3H, *P < 0.05, **P < 0.01). These results were consistent with the sequencing results.

**Table 2:** Primer sequences used for gene amplification in the rat model

Primer names	Forward sequences ( 5′-3′)	Reverse sequences ( 5′-3′)
β-actin	tggaatcctgtggcatccatgaac	taaaacgcagctcagtaacagtccg
Hspa1a	gagggtctcaagggcaagat	ttctcagccagcgtgttaga
Hspa1b	tctaacacgctggctgagaa	agcagccatcaagagtctgt
Hspb1	acgaagaaaggcaggatgaa	gacagggaagaggacaccag
Atf6	cccaccaaaggtcagactgt	cttgggactttgagcctctg
Chop2	agcagaggtcacaagcacct	acagttcatgcttggtgcag
Wfs1	cagccgagaagggacagata	ggcaagtcgcaggtagtgtt
Calreticulin	gaatggaagccacgtcaaat	tggcctctacagctcatcct
Atg3	cggtcctcaaggaatcaaaa	gcctccaattcatccgaata
Atg12	aagatggcagaagacccaga	tgaagtcgatgagtgcttgg
Bcl-2	aagctgtcacagaggggcta	ctcacttgtggcccaggtat
Bax	ctgcagaggatgattgctga	gatcagctcgggcactttag
Caspase-12	gctgccaagagaacacatga	tccctttgcttgtggatacc
Canx	gatgctgtcaagccagatga	ttagggttggcaatctgagg
Cacna1e	gcccttaaagctcgtgtcag	gcattggaagacagtcagca
Slc24a2	ccgaggaagatgatgaccag	tccagagagggaacacgatg
Ttr	actggaaggctcttggcatt	gctgtaggagtacgggctga

**Table 3:** Primer sequences used for gene amplification in the cell model

Primer names	Forward sequences ( 5′-3′)	Reverse sequences ( 5′-3′)
β-actin	acactgtgcccatctacgagggg	atgatggagttgaaggtagtttcgtggat
Hspa1a	gatcaacgacggagacaagc	gctgcgagtcgttgaagtag
Hspa1b	gatcaacgacggagacaagc	gctgcgagtcgttgaagtag
Hspb1	ccaagtttcctcctccctgt	ggtgactgggatggtgatct
Atf6	gcagaacctcagccactttc	gctgctggaagcaataaagg
Chop2	tcttgaccctgcttctctgg	gctgtgccactttcctttca
Wfs1	ccctacctggtgtgcttcat	gagctccagagacgtgaacc
Calreticulin	ccccagtgattcagaaccct	gccaaactcctcagcgtatg
Atg3	acggcagcctttaacagttg	cttcatcacctcagcatgcc
Atg12	ctggaggggaaggacttacg	gggaaggagcaaaggactga
Bcl-2	gggtacgataaccgggagat	ctgaagagctcctccaccac
Bax	ggggacgaactggacagtaa	cagttgaagttgccgtcaga
Caspase-12	caagcactgggatcaagag	aaccaaacaatcccagcacc

**Figure 4:**
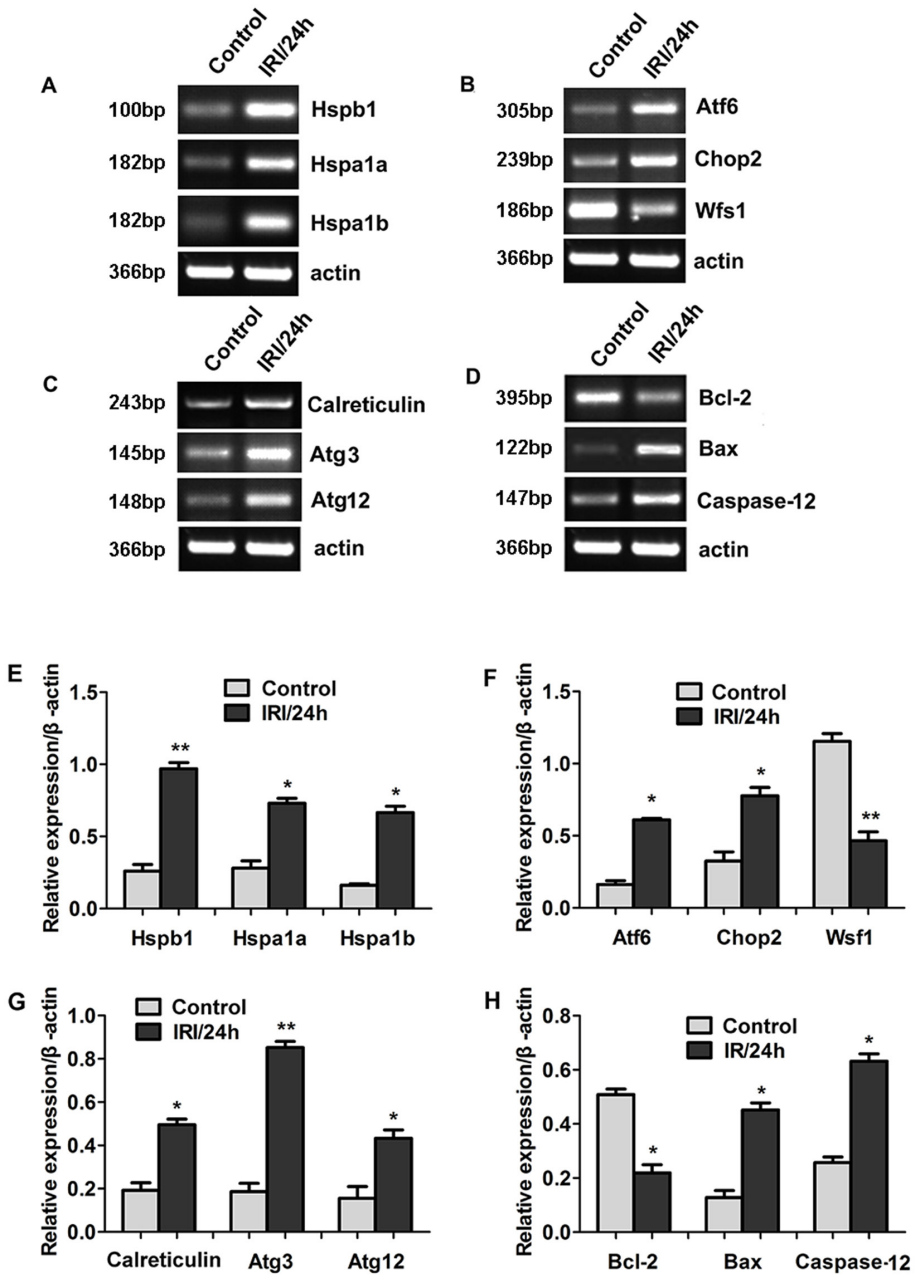
Differential gene expression between OGD cell model and control was detected by RNA sequencing (Panel **A-D**) Representative images of RT-PCR confirming the differential gene expression. (Panel **E-H**) Quantitative analysis of gene expression. *P < 0.05, **P < 0.01 *vs* control.

To understand the expression of the above genes in the OGD cell model, we compared their expression between the OGD model and the control model. As shown in Figure 4, the expression trends of these genes in the OGD model were consistent with the trends in the animal models. The up-regulated genes were Hspb1, Hspa1a, Hspa1b, Atf6, Chop2, calreticulin, Atg3, Atg12, Bax, and Caspase-12. The down-regulated genes were Wfs1 and Bcl-2. The results obtained using RT-PCR were consistent with the results obtained by RNA sequencing.

### Gene expression patterns vary over the course of IRI

**Figure 5:**
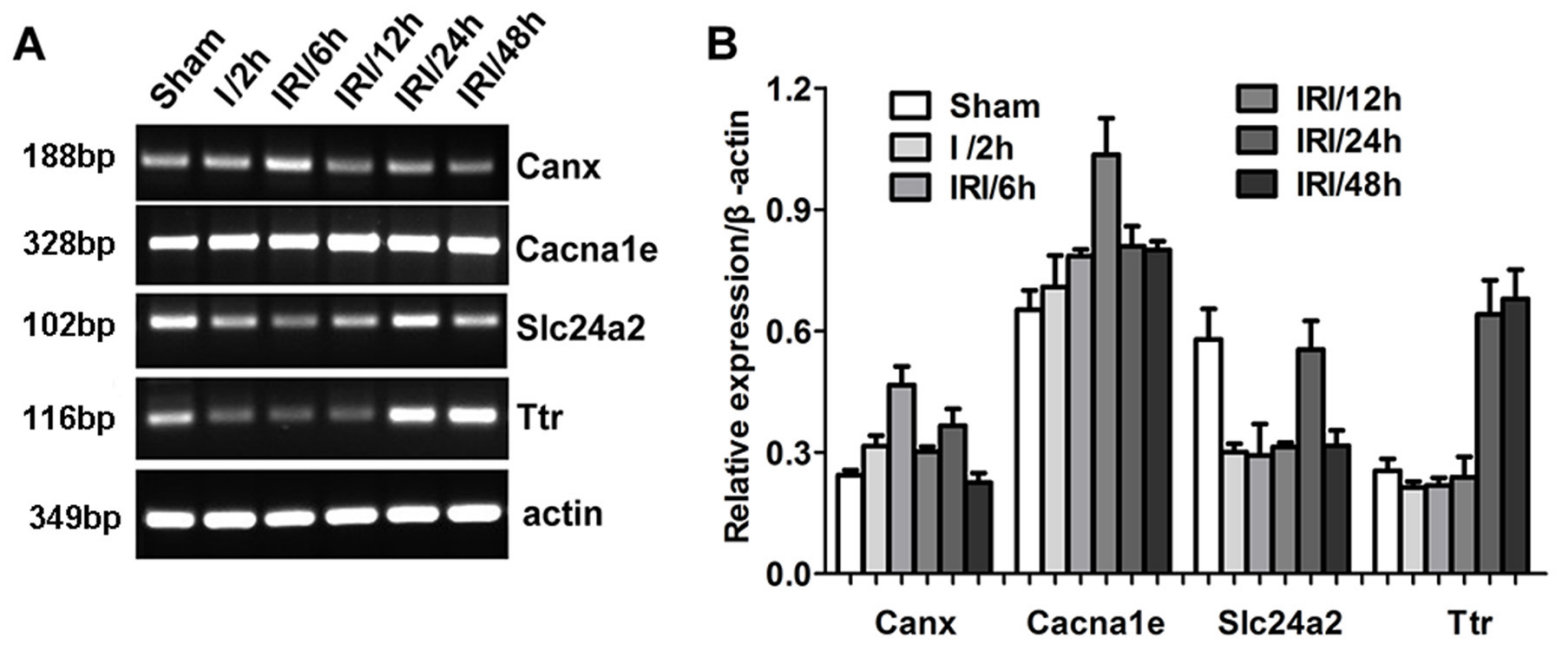
Differential gene expression at different reperfusion times was detected by RNA sequencing (Panel **A**) Representative images of RT-PCR demonstrating differential gene expression. (Panel **B**) Quantitative analysis of gene expression.

In addition, we detected the expression of several genes at different times post-reperfusion. As shown in Figure 5, the expression patterns of four genes displayed significant differences at various times during the post-reperfusion period. The expression of the Canx gene was highest at 6 h post-reperfusion, and then gradually decreased. The Cacna1e gene reached its highest expression levels at 12 h post-reperfusion, then decreased. The expression of Slc24a2 first decreased, then gradually increased, peaked 24 h post-reperfusion, then decreased once more. Ttr gene expression was reduced during the first 12 h post-reperfusion, but was dramatically raised at 24 h and 48 h post-reperfusion. These results demonstrate that the patterns of gene expression are complex and diverse, increasing the difficulty of understanding the molecular pathology of IRI.

### Elevation of TNF-α, IL-6, and Icam-1 levels in rat and cell models

**Figure 6:**
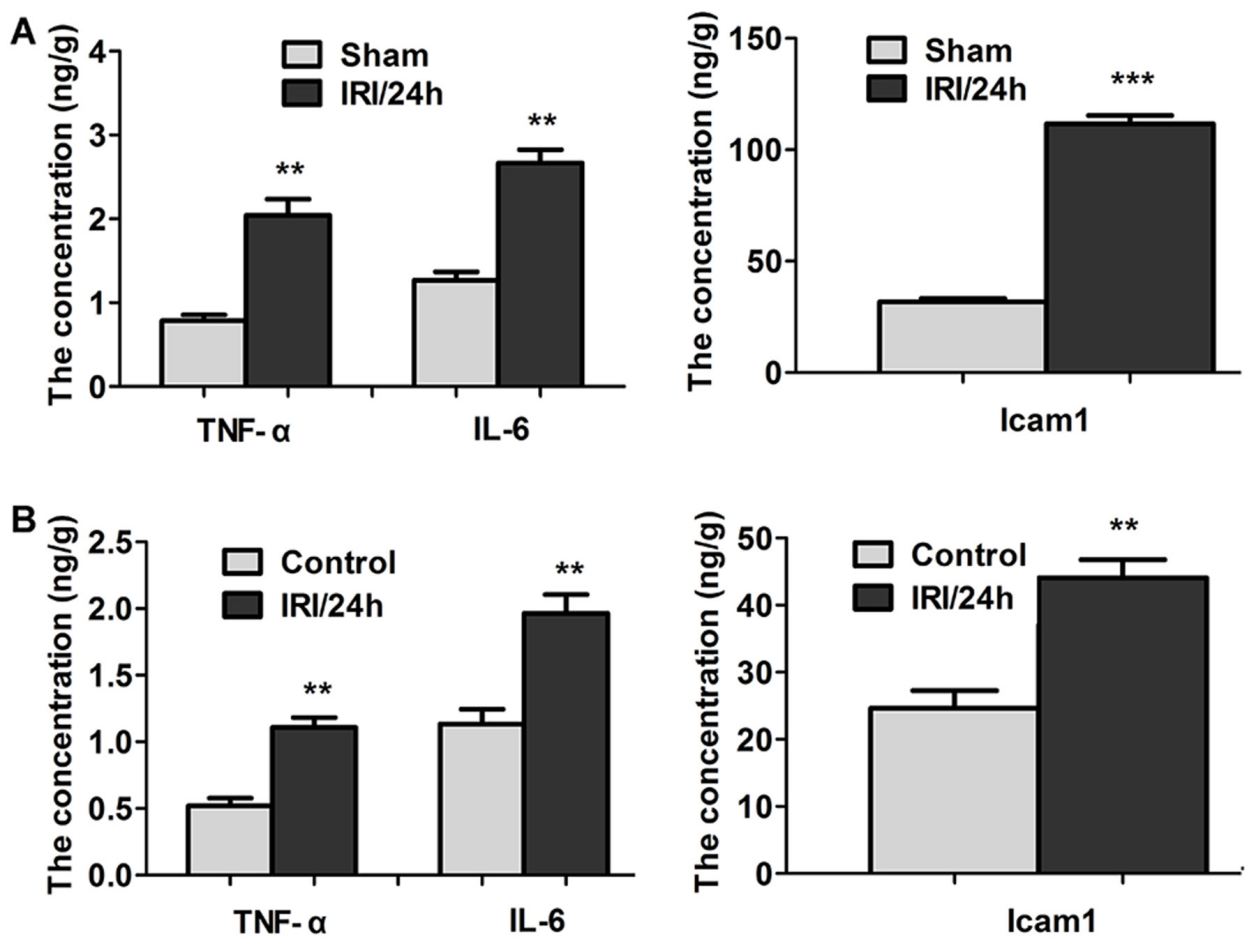
TNF-α, IL-6, and ICAM-1 levels were detected by ELISA (Panel **A**) TNF-α, IL-6, and ICAM-1 levels in hippocampi of sham and IRI/24 h groups. (Panel **B**) TNF-α, IL-6, and ICAM-1 levels in the control and the OGD cell model. The levels of TNF-α, IL-6, and ICAM-1 were significantly increased in the ischemia-reperfusion group compared with the sham group. **P < 0.01, ***P < 0.001.

The expression of proinflammatory factors and adhesion molecules was detected using ELISA. As shown in Figure 6A, the levels of TNF-α, IL-6, and ICAM-1 in the hippocampi of the IRI/24 h group were all higher than the levels in the hippocampi of the sham group. TNF-α was increased by 2.62-fold (2.04 ± 0.42 vs 0.78 ± 0.07 ng/g), IL-6 by 2.86-fold (2.66 ± 0.16 vs 0.93 ± 0.41 ng/g), and Icam-1 by 3.95-fold (111.72 ± 3.69 vs 28.29 ± 1.64 ng/g). The levels of TNF-α, IL-6, and ICAM-1 were also detected in the OGD cell model (Figure 6B). TNF-α, IL-6, and ICAM-1 were up-regulated with fold increases of 2.13 (1.11 ± 0.07 vs 0.52 ± 0.06 ng/g), 1.73 (1.96 ± 0.14 vs 1.13 ± 0.11 ng/g), and 1.79 (44.08 ± 2.71 vs 24.63 ± 2.63 ng/g), respectively, compared with the sham group. These findings indicate that reperfusion following ischemic injury promotes the activation of inflammatory factors and adhesion molecules.

### GO analysis of biological functions of differentially expressed genes

**Figure 7:**
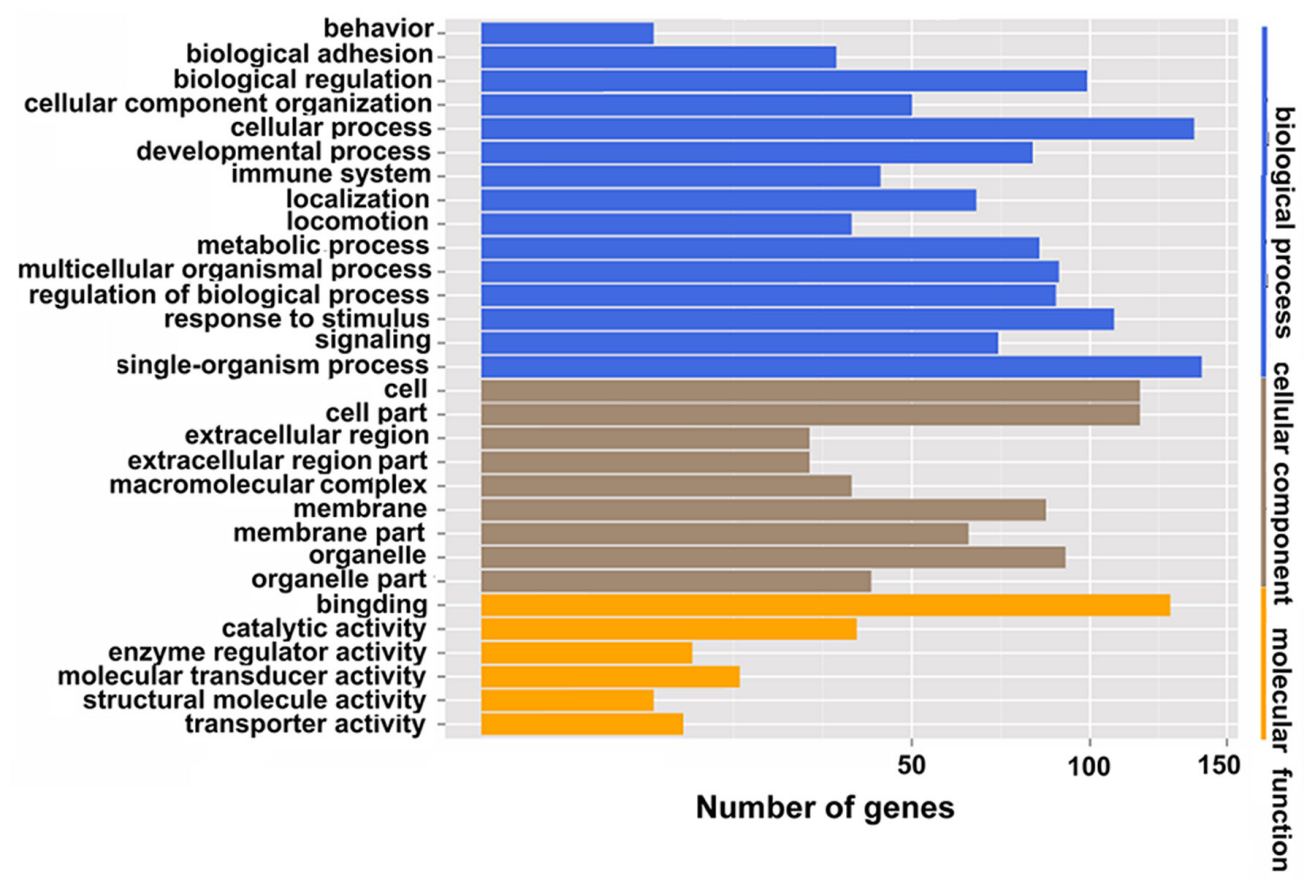
GO functional classification of IRI and sham groups Blue denotes biological process; brown denotes cellular component; yellow denotes molecular function.

To elucidate the roles of differentially expressed genes, GO analysis was used to analyze the biological functions of those genes. As shown in Table 4 and Figure 7, the differentially expressed genes were divided into three functional groups. In the biological process group, the top four categories were single-organism processes, cellular processes, response to stimulus, and biological regulation. The genes belonging to these categories were Atf3, Ssc5d, Lgals3, Olr1, Sema3d, Pter, Myd88, Selplg, and Wfs1. They are involved in responding to various stimuli, metabolic processes, the inflammatory response, and cell signaling. In the molecular function group, the identified categories were binding and activation. Genes belonging to this category were Igfbp2, Fibcd1, Socs3, Gfap, Pla1a, and Cd3e. They are involved in growth factor binding, carbohydrate derivative binding, protein kinase regulator activity, structural molecule activity, catalytic activity, and signaling receptor activity. The cellular components group categories included cell, cell part, membrane components, and organelles. The genes Abca4, Tspo, and Siglec5 belonged to the membrane components category. These results suggest that IRI induces abnormalities of biological processes, molecular function, and cellular components.

**Table 4:** Gene ontology classification of differentially expressed genes

GO term	Input symbol	Expression	P-value
**Biological process**			
GO:0050896//response to stimulus	Atf3	up	5.20E-08
GO:0006950//response to stress GO:0019538//protein metabolic process	Ssc5d Olr1	up up	6.23E-16 6.20E-06
GO:0006952//defense response	Lgals3	up	4.12E-06
GO:0006954//inflammatory response	Myd88	up	6.79E-07
GO:0048869//cellular developmental process	Sema3d	up	8.52E-06
GO:0007154//cell communication	Nts	down	1.15E-26
GO:0006984//ER-nucleus signaling pathway GO:0007159//leukocyte cell-cell adhesion	Wfs1 Selplg	down down	2.06E-07 5.65E-14
GO:0008152//metabolic process	Pter	down	6.51E-10
**Molecular function**			
GO:0019887//protein kinase regulator activity	Socs3	up	6.07E-12
GO:0005198//structural molecule activity	Gfap	up	0
GO:0003824//catalytic activity GO:0005216//ion channel activity	Pla1a Piezo1	up up	1.05E-07 3.45E-08
GO:0005520//insulin-like growth factor binding	Igfbp2	up	2.03E-11
GO:0038023//signaling receptor activity	Cd3e	down	6.23E-08
GO:0097367//carbohydrate derivative binding	Fibcd1	down	1.53E-31
**Cellular components**			
GO:0032991//macromolecular complex GO:0043229//intracellular organelle	Tubb6 Slc1a4	up up	6.83E-23 1.72E-09
GO:0031966//mitochondrial membrane GO:0042101//T cell receptor complex	Tspo Cd3e	up down	5.40E-06 6.23E-08
GO:0031224//component of membrane	Siglec5	down	4.08E-06

### Pathway analysis of differentially expressed genes

**Figure 8:**
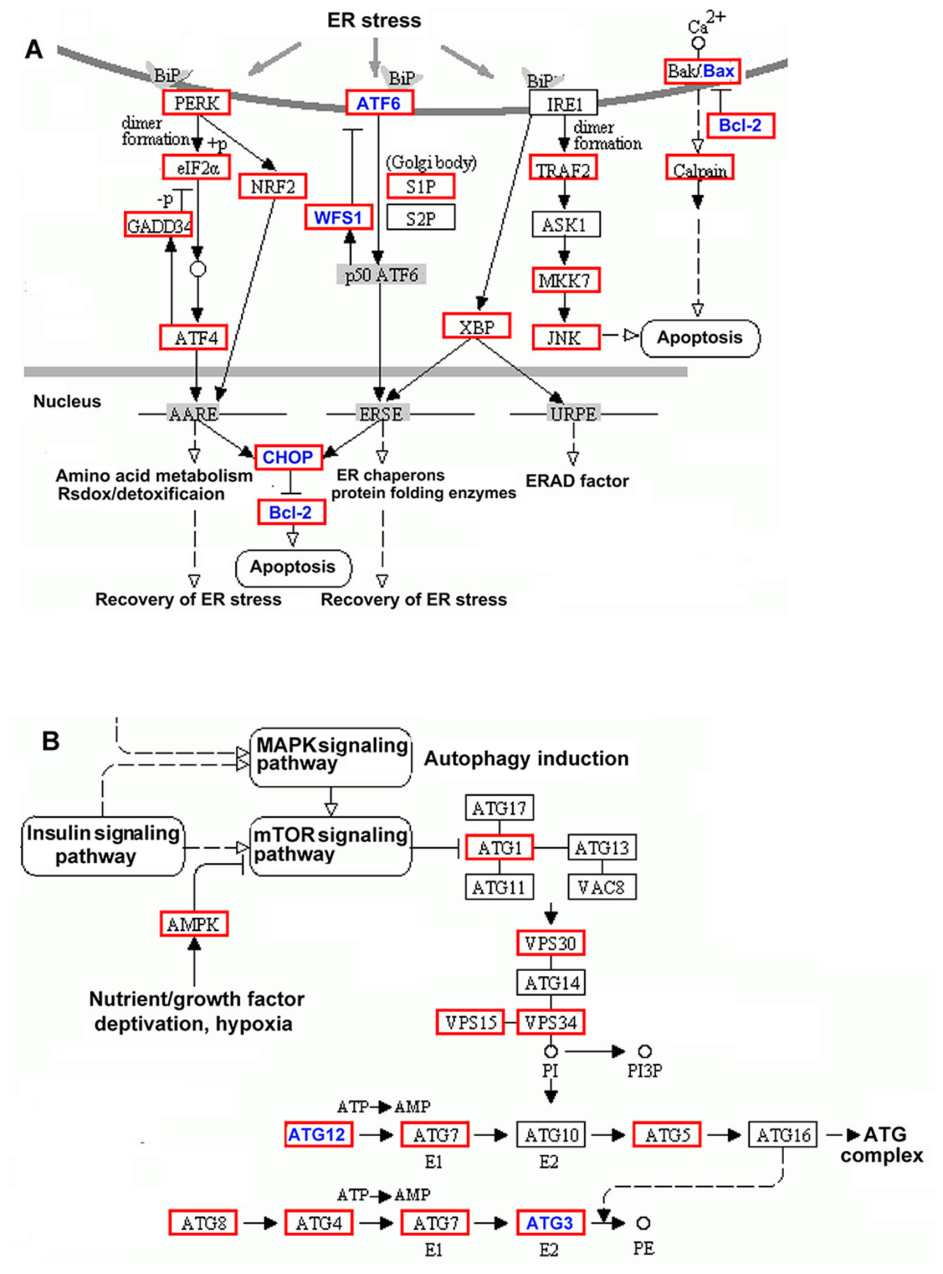
KEGG pathway classification of differentially expressed genes (Panel **A**) ER stress signaling pathway. Blue denotes differentially expressed genes in ER stress signaling pathways. (Panel **B**) Autophagy pathway. Blue denotes differentially expressed genes in autophagy pathways.

KEGG pathway analysis was used to provide a more in-depth understanding of the biological functions of the differentially expressed genes. Pathways that were significantly induced after cerebral IRI are listed in Table 5. This analysis suggests that the cellular pathways involved in IRI include focal adhesion, regulation of the actin cytoskeleton, cytokine-cytokine receptor interaction, MAPK signaling, Jak-STAT signaling, cell adhesion molecules, and ER stress. Figure 8 shows two significantly induced pathways, ER stress signaling and autophagy. In the ER stress signaling pathway, the genes ATF6, WFS1, CHOP2, Bcl2, and Bax were differentially expressed in the IRI/24 h group, as detected by RNA sequencing. In the autophagy pathway, the expression of ATG3 and ATG12 was significantly increased at 24 h post-reperfusion. These results suggest that signaling pathways containing different genes were actived or inhibited in the process of IRI.

**Table 5:** KEGG pathway classification of differentially expressed genes

Pathway	Genes	P-value	Pathway ID
Focal adhesion	Itga, Dock1, Rock, Rtk	2.094476e-05	ko04510
Regulation of actin cytoskeleton	Pxn, Rock, Itg, Limk, Arp2/3	0.002440363 0.0009153268	ko04810 ko04060
Cytokine-cytokine receptor interaction	Lxcl16, cl2, Sf1a	0.0009153268	ko04060
Phagosome	Tubb, MhcII, αMβ2	0.001802472	ko04145
MAPK signaling pathway	Fgfr, Hsp72, c-fos	0.001802472	ko04010
Jak-STAT signaling pathway	STAT, Myc, Socs	0.001047253	ko04630
Cell adhesion molecules	Itgb2, Selplg, Ptprf	0.001865349	ko04514
Toll-like receptor signaling pathway	Lbp, Opn, Myd88	0.001876453	ko04620
ER stress	Atf6, Chop, Wfs1	0.003695505	ko04141

## DISCUSSION

The MCAO is a universal model used to establish a focal cerebral IRI without the need for a craniotomy operation. This model enables accurate control of ischemia-reperfusion time. Previous experiments in our lab confirmed other reports [12] that transient cerebral ischemic injury for 2 h followed by post-reperfusion measurements at 24 h provides the most appropriate model of IRI. Accordingly, 2 h of ischemia followed by analysis of hippocampi sampled at 24 h post-reperfusion were the conditions under which abnormal expression of genes was evaluated.

It has been previously established that genes related to inflammation, apoptosis, proliferation, cell cycle, ER stress, and autophagy [13–16] are involved in the cellular events observed in cerebral IRI. Our results obtained from RNA sequencing were similar to those of previous studies, and draw a consistent conclusion regarding the genes involved in IRI.

The expression of inflammatory factors was increased in the pathological process of cerebral IRI. The release of inflammatory molecules, including TNF-α, IL-1β, IL-6, NF-κB, and TGF-β, likely further aggravates cell injury during ischemia-reperfusion [17, 18]. Consistent with previous studies in rat and cell models, IRI significantly induced the increased expression of TNF-α, IL-6, and intercellular adhesion molecule (ICAM1), indicating that the inflammatory response was actived by cerebral IRI. Specific molecules that bind neutrophils, including CAMs and selectins (ICAM2, ICAM4, ICAM5, VCAM, selectin E, and selectin L), exhibited abnormal expression in our experiment. These genes play an important role in leukocyte infiltration and transmigration [19]. Gfap is a strong marker of gliosis, and may also serve as a marker of neuroinflammation [20, 21]. In the current study, the expression of Gfap was up-regulated 2.286-fold (Table 3). This suggests that the over-activation of glia may be induced during IRI.

Cellular apoptosis may accompany cerebral IRI due to the expression of apoptosis-related genes [22, 23]. Bcl-2, a key antiapoptotic factor, could inhibit the release of cytochrome c. Bax, as a proapoptotic factor, may increase the release of cytochrome c. Various reports suggest that Bcl-2 expression is decreased and Bax expression is increased at different reperfusion times [24, 25]. Consistent with these results, we demonstrated that Bcl-2 expression was decreased and Bax expression was increased in the hippocampus at 24 h post-reperfusion, as measured by RNA sequencing and RT-PCR. The differential expression of other genes related to apoptosis, including Bad, Bcl-6, Bak, Bcl2-like 1-15 (apoptosis facilitator), Bcl2-associated factors, and Bag (BCL2-associated athanogene), was detected through RNA sequencing. The expression of Caspase family members was also altered. Caspase-4, Caspase-7, and Caspase-8 exhibited 4.19-, 2.19-, and 1.57-fold increases, respectively, indicating that these genes may involve in IRI.

Hsps are ubiquitous and highly conserved proteins that are induced in response to stressors including high temperature, ischemia, hypoxia, and pathological state [26, 27]. Hsps may be induced after cerebral ischemia, and their expression may protect against cellular damage via anti-inflammatory and antiapoptotic mechanisms, or the repair of damaged molecules [28–30]. In the present study, Hspb1, Hspa1a, and Hspa1b were significantly increased at 24 h post-reperfusion in cellular and rat models. It is essential to further research the function of these genes to fully understand the mechanism of IRI.

Wolfram syndrome 1 (Wfs1) and activating transcription factor 6α (Atf6α) are transmembrane proteins localized to the ER [31, 32]. Wfs1 negatively regulates Atf6α through the ubiquitin-proteasome pathway. Wfs1 stabilizes the E3 ubiquitin ligase HRD1, leading to the suppression of ER stress signaling. In the current study, we further verified the inverse relationship between Wfs1 and Atf6. The expression level of Wfs1 was significantly decreased, but Atf6 and Chop2 expression levels were significantly increased. This indicates that cerebral IRI induces a decrease in Wfs1 expression, which in turn activates the Atf-Chop pathway (Figure 8A). Previous reports [24] demonstrated that extended periods of ER stress activate Caspase-12, leading to cell death. Our results confirm these reports. Expression of Caspase-12 increased 3.59-fold based on RNA-sequencing analysis, and 2.85-fold based on RT-PCR analysis. These results suggest that ER stress is involved in cerebral IRI.

Previous reports established a connection between cerebral IRI and the activation of autophagy [33, 34]. Autophagy is a conserved homeostatic process that maintains cellular balance through the lysosomal system [35, 36]. In normal physiological processes, misfolded molecules or damaged organelles are eliminated through autophagy. However, non-apoptotic programmed cell death is initiated through excessive self-digestion and degradation of cellular components if autophagy is activated inappropriately [37, 38]. Both cerebral ischemia and ischemia-reperfusion can activate autophagy by inducing high expression of certain genes, including Becn1, LC3-II, and Atg5 [39, 40]. To assess the level of autophagy after cerebral ischemia-reperfusion, we carried out global gene profiling using RNA sequencing. We found abnormal levels of calreticulin, Atg3, Atg12, Becn1, Atg5, and Ukl1. Calreticulin, Atg3, and Atg12 were selected for amplification using RT-PCR. Consistent with the results obtained from RNA sequencing, these genes were dramatically elevated 24 h post-reperfusion. These results suggest that autophagy was over-activated by cerebral IRI.

Reperfusion after ischemic injury may give rise to two openings of the blood-brain barrier (BBB) during the refractory period [41]. The initial opening phase occurs several hours post-reperfusion and is transient. An irreversible second phase opening occurs 24 h to 72 h post-reperfusion. Consistent with this pathological phenomenon, a therapeutic strategy for ischemic stroke should be carried out as early as possible. In the present study, Canx, Cacna1e, Slc24a2, and Ttr were selected, and their expression levels were examined at 6, 12, 24, and 48 h after reperfusion. The expression of these genes was dynamic during the 48 h following reperfusion, and the peak expression time of each gene was not identical. This suggests that these genes play different role in IRI at different times following ischemia-reperfusion, underscoring the idea that the pathological processes of IRI are complex and diverse.

In summary, a global gene expression profile for the hippocampus after cerebral IRI was obtained using RNA sequencing. 156 genes were up-regulated and 26 genes were down-regulated. GO analysis and KEGG pathway analysis suggested that differentially expressed genes were involved in inflammation, oxidative stress, ER stress, apoptosis, autophagy, and metabolism. This study provides new insights into understanding the complicated molecular mechanisms of cerebral IRI. Further elaboration of the differentially expressed genes and relevant pathways will be beneficial for the prevention and treatment of cerebral IRI.

## MATERIALS AND METHODS

### Experimental animals

Male Wistar rats, aged 6–8 weeks, weighing 230–280 g, were purchased from Pengyue Experimental Animal Breeding Institute of Jinan, China (license no. SCXK (Lu) 2014-0007). The rats were housed in a controlled environment of 22–26°C, 50–60% humidity, and a 12 h light/dark cycle. All animal experiments were approved by the Ethics Committee of Jining Medical University and performed in accordance with the international Health Guide for the Care and Use of Laboratory Animals.

### Middle cerebral artery occlusion (MCAO) model

The rat middle cerebral artery occlusion (MCAO) model was established as follows: Rats were anesthetized using 10% chloral hydrate (0.3 mL/100 g) through intraperitoneal injection, and fixed in the supine position. The right common carotid artery was isolated, and a 0.26 mm diameter fishing line was inserted along the right common carotid arteries into the internal carotid artery until it blocked the origin of the middle cerebral artery. The depth of penetration was approximately 18 mm. The incision was sutured. According to the experimental protocol of our lab [10, 42], rats were subjected to focal ischemia for 2 h, and the fishing lines were pulled out post-reperfusion at 2, 6, 12, 24, and 48 h. In the sham group, the right carotid artery was isolated without occlusion of the common carotid arteries. The surgery was performed at 22 ± 2°C. A neurological score was used to evaluate the success of the model. After the neurological score was obtained, the qualifying rats in each group were decapitated, and their hippocampi were isolated and stored on ice for use in the experiments described below.

### 2, 3, 5-Triphenyl-2H-tetrazolium chloride (TTC) staining

Brains were isolated from the 24 h post-reperfusion (IRI/24 h) group and the sham group. Brains were sliced into 2-mm-thick coronal sections. Tissue sections were incubated for 30 min at 37°C in 1% 2, 3, 5-triphenyl-2H-tetrazolium chloride (TTC) solution (Sigma, St. Louis, MO, USA) in the dark. Slices were then collected for imaging. Infarct volume was determined using Image-Pro Plus v 6.0 software. Infarct volume ratio was calculated using the following formula: Infarct volume ratio = (Σ infarct area × thickness) / (Σ brain slices area × thickness) × 100%.

### RNA extraction and sequencing

Total RNA from the IRI/24 h and sham groups was extracted using Trizol reagent (Invitrogen, Carlsbad, USA) and treated with DNase I to degrade contaminating DNA. The quality and concentration of the RNA was evaluated using agarose gel electrophoresis and the BioSpec-nano spectrophotometer (Shimadzu, Japan). Total mRNA was then isolated using oligo (dT), and fragmented into short fragments. First and second strand cDNA synthesis was carried out on individual mRNAs. Double-stranded cDNA was purified and modified with end repair, 3’-end single nucleotide adenine (A) addition, and sequencing adaptors. Finally, the fragments were enriched by PCR amplification to construct a library ready for sequencing using Illumina HiSeq TM 2000 in Shenzhen Huada Genetics Corporation (Shenzhen, China).

### Bioinformatics analysis

Primary sequencing data produced by RNA sequencing, called raw reads, were subjected to quality control (QC). After QC, raw reads were filtered into clean reads. QC of alignment was performed to determine if re-sequencing was needed. If the alignment result passed QC, downstream analysis including gene expression, differentially expressed genes, cluster analysis, Gene Ontology (GO) enrichment analysis, and pathway enrichment analysis was proceeded with.

### Cluster analysis

Distances of expressed genes were calculated using the Euclidean method. The sum of the squared deviations algorithm was used to calculate distance. The cluster analysis of gene expression patterns was performed using java Treeview software.

### Screening of differentially expressed genes

To identify differentially expressed genes when comparing the IRI/24 h and sham groups, the Bonferroni correction was performed on P-values. The thresholds required for the results to be considered significant were as follows: FDR ≤0.001 and absolute value of log^2^ Ratio ≥1.

### Semi-quantitative PCR

The extracted total RNA was reverse-transcribed into cDNA using the FastQuant RT kit (Tiangen Biotech, Beijing, China). The cDNAs were then used in amplification reactions with 2× Taq PCR Master Mix (Tiangen Biotech, Beijing, China). β-actin was used as an internal reference gene. The reaction parameters were as follows: 94°C for 3 min, followed by 30 cycles at 94°C for 40 sec, 55°C for 50 sec, and 72°C for 50 sec. The amplification products were separated by electrophoresis on a 1% agarose gel, and analyzed using ImageJ2x software.

### Oxygen-glucose deprivation (OGD) model

SHY-5Y cells were purchased from the Cell Resource Center of the Chinese Academy of Sciences (Shanghai, China). Cells were grown in DMEM medium supplemented with 10% FBS, 100 U/mL penicillin, and 100 μg/mL streptomycin in a humidified atmosphere of 5% CO_2_ at 37°C. For the oxygen-glucose deprivation (OGD) model, SHY-5Y cells were divided into two groups: (1) Control group: Cells were cultured in DMEM without OGD; (2) OGD model group: Cells were incubated in Eagle’s medium for 2 h at 37°C in an incubator under 5% O_2_, 5% CO_2_, and 90% N_2_. Cells were then cultured in fresh medium for 24 h at 37°C in an incubator under 5% CO_2_.

### Enzyme-linked immunosorbent assay (ELISA)

Homogenates of hippocampi from IRI/24 h and sham groups were made and centrifuged at 4°C. The supernatants were collected, and used to detect the levels of TNF-α, IL-6, and ICAM-1 using enzyme-linked immunosorbent assay (ELISA) kits from Cloud-Clone Corp. (Wuhan, China), according to the manufacturer’s instructions. All ELISA analyses were carried out in triplicate. The concentrations of TNF-α, IL-6, and ICAM-1 were determined according to a standard curve.

### GO enrichment analysis

GO enrichment was used to note the function of differentially expressed genes via the GO database (http://www.geneontology.org/). A Bonferroni correction was used to obtain a corrected P-value. GO terms with P-values ≥0.5 were regarded as significantly enriched in the target candidates. After obtaining GO annotation for differentially expressed genes, WEGO software was used for GO functional classification of differentially expressed genes [43].

### **Kyoto** encyclopedia of genes and genomes (KEGG) pathway analysis

The Kyoto Encyclopedia of Genes and Genomes (KEGG) pathway database (http://www.genome.jp/kegg/) was used to determine pathway enrichment of differentially expressed genes compared with the reference gene background. Genes with FDR ≤0.05 were regarded as significantly enriched. In addition, a scatter plot for KEGG enrichment results was generated.

### Statistical analysis

All data were presented as mean ± SEM and analyzed using GraphPad Prism 5.0 software. Significance was determined using Student’s t-test. Differences between group means with *P* < 0.05 were considered statistically significant.
